# Dynamics of human monocytes and airway macrophages during healthy aging and after transplant

**DOI:** 10.1084/jem.20191236

**Published:** 2020-01-09

**Authors:** Adam J. Byrne, Joseph E. Powell, Brendan J. O’Sullivan, Patricia P. Ogger, Ashley Hoffland, James Cook, Katie L. Bonner, Richard J. Hewitt, Simone Wolf, Poonam Ghai, Simone A. Walker, Samuel W. Lukowski, Philip L. Molyneaux, Sejal Saglani, Daniel C. Chambers, Toby M. Maher, Clare M. Lloyd

**Affiliations:** 1 National Heart and Lung Institute, Imperial College London, London, UK; 2 Asthma UK Centre in Allergic Mechanisms of Asthma, London, UK; 3 Garvan-Weizmann Centre for Cellular Genomics, Garvan Institute of Medical Research, Darlinghurst, Sydney, Australia; 4 Cellular Genomics Futures Institute, University of New South Wales, Kensington, Sydney, Australia; 5 Queensland Lung Transplant Service, The Prince Charles Hospital, Brisbane, Queensland, Australia; 6 Faculty of Medicine, The University of Queensland, Brisbane, Queensland, Australia; 7 National Institute for Health Research Respiratory Biomedical Research Unit, Royal Brompton Hospital, London, UK; 8 Institute for Molecular Bioscience, The University of Queensland, Brisbane, Queensland, Australia

## Abstract

The ontogeny of airway macrophages (AMs) in human lung and their contribution to disease are poorly mapped out. In mice, aging is associated with an increasing proportion of peripherally, as opposed to perinatally derived AMs. We sought to understand AM ontogeny in human lung during healthy aging and after transplant. We characterized monocyte/macrophage populations from the peripheral blood and airways of healthy volunteers across infancy/childhood (2–12 yr), maturity (20–50 yr), and older adulthood (>50 yr). Single-cell RNA sequencing (scRNA-seq) was performed on airway inflammatory cells isolated from sex-mismatched lung transplant recipients. During healthy aging, the proportions of blood and bronchoalveolar lavage (BAL) classical monocytes peak in adulthood and decline in older adults. scRNA-seq of BAL cells from lung transplant recipients indicates that after transplant, the majority of AMs are recipient derived. These data show that during aging, the peripheral monocyte phenotype is consistent with that found in the airways and, furthermore, that the majority of human AMs after transplant are derived from circulating monocytes.

## Teaser

We characterized monocyte/macrophage populations from infants to older adults and lineage traced these cells using sex-mismatched lung transplant recipients. Classical monocytes peak in adulthood and decline in older adults. Importantly, our data show that after transplant, donor human airway macrophages (AMs) are readily replaced by recipient-derived monocytes.

## Introduction

Airway macrophages (AMs) are the most abundant immune cell type present in the airspace under homeostatic conditions and are strategically positioned to aid in lung defense (Byrne et al., 2015, 2016). Recent work in mice has indicated that many tissue-resident macrophages, including those found in the lung, self-maintain locally, with minimal contribution from circulating monocytes, during steady-state conditions (Schulz et al., 2012; Guilliams et al., 2013; Hashimoto et al., 2013; van de Laar et al., 2016; Svedberg et al., 2019). During pulmonary inflammatory responses in mice, it has been shown that monocytes are recruited to the lung and in response to local cues develop into AM-like cells (Hashimoto et al., 2013; Gibbings et al., 2015). However, our understanding of AM ontogeny, aging, and contribution to disease is largely based on murine models, with their attendant limitations in key factors such as environmental exposures and life span. It is critical to understand these processes in the human lung in order to elucidate the contribution that AM populations make to both healthy aging and pathogenesis of lung diseases.

The circulating monocyte pool in humans consists of multiple subsets that may be distinguished based on the expression of CD14 and CD16. CD14^+^CD16^−^ classical monocytes (CMs) make up the majority of the circulatory population, whereas the remaining pool consists of CD14^+^CD16^+^ intermediate monocytes (IMs) and CD14^lo^CD16^+^ nonclassical monocytes (NCMs; Yona et al., 2013). CMs or inflammatory monocytes circulate in the blood and egress into tissues after injury (Zigmond et al., 2014) or infection (Serbina and Pamer, 2006) and subsequently differentiate into mature macrophage populations. In contrast, IMs patrol the luminal surface of small vessels and play a key role in immune surveillance (Auffray et al., 2007).

In rodents, AMs differentiate shortly after birth and persist over the murine life span via self-renewal, with minimal contribution from circulating CMs (Hashimoto et al., 2013). AMs maintain homeostasis in the lung through reciprocal cell–cell and soluble mediator interactions with the airway epithelium. This creates a regulatory environment that limits unwarranted inflammatory responses (Hussell and Bell, 2014). When this regulatory milieu is breached, circulating monocytes are recruited to the airway lumen, where they differentiate into AMs and orchestrate proinflammatory and profibrotic responses (Byrne et al., 2015; Osterholzer et al., 2013; Moore et al., 2001). Thus, the injured murine lung contains at least two ontologically distinct AM populations. However, it is unclear how this model of macrophage ontogeny applies to the human lung and whether perinatally derived AMs are present in adult airways, which have encountered a lifetime of inhaled exposures. Indeed environmental exposures have been shown to desensitize murine AM populations and reduce responsiveness (Didierlaurent et al., 2008).

Here, we describe the circulating monocyte pool, as well as resident AM populations, through the human life span, from early life (2–12 yr) to adulthood (20–50 yr) and in older adults (>50 yr). We found that the activation pattern of circulating CMs throughout life is comparable to those found in the airways, suggesting ongoing recruitment to the lung from CM precursors. Using bronchoalveolar lavage (BAL) samples from sex-mismatched lung transplant patients, we show that the majority of AMs in the human lung after transplant are recipient derived. Together, this work highlights the critical role of AMs of peripheral origin in human pulmonary health.

## Results and discussion

### Patient characteristics

In all, 42 healthy subjects were enrolled in the present study. Volunteers were aged 20–50 yr (*n* = 8; 26 ± 6 yr) and >50 yr (*n* = 11; 58 ± 4 yr). Pediatric controls underwent a clinically indicated bronchoscopy and were aged 2–12 yr (*n* = 23; 5 ± 2 yr). Demographic and clinicopathological features for healthy subjects are shown in Table 1, and transplant patients are shown in Table 2. Subjects included in the study had normal lung function and no history of pneumonia, intensive care unit stays, or other hospitalizations for respiratory concerns.

**Table 1.** Clinical characteristics of healthy children or adult volunteers included in this study

	Children	Younger adults	Older adults
**Number**	23	8	11
**Age (yr), mean ± SD**	5 ± 2	26 ± 6	58 ± 4
**Sex (female, male)**	12, 11	5, 3	4, 7
**Smoking (ever, never, current)**	N/A	0, 6, 2	1, 7, 3
**FVC (% predicted, ± SD)****a**	N/A	97.84 ± 9	102.65 ± 9.4
**BAL cell count/ml (×10^6^)****a**	0.11 ± 0.1	3.28 ± 2	2.27 ± 1.26

FVC, forced vital capacity; N/A, not applicable.

a Data from adult volunteers or children undergoing bronchoscopy only.

**Table 2.** Donor/recipient origin of AMs in transplant patients

Age (yr)	Sex	Sex (donor)	Indication	Time after TX (mo)	% XIST^+^	% RPS4Y1^+^
59	M	F	IPF	8.4	10.48	59.73
20	F	M	CF	20.7	70.08	0.54
58	F	M	COPD	55.2	55.84	0.32
46	F	M	CF	112.8	72.88	0.19

CF, cystic fibrosis; COPD, chronic obstructive pulmonary disease; F, female; IPF, idiopathic pulmonary fibrosis; M, male; TX, transplant.

### CM populations in the periphery are similar to those found in the airway

**Figure 1.**
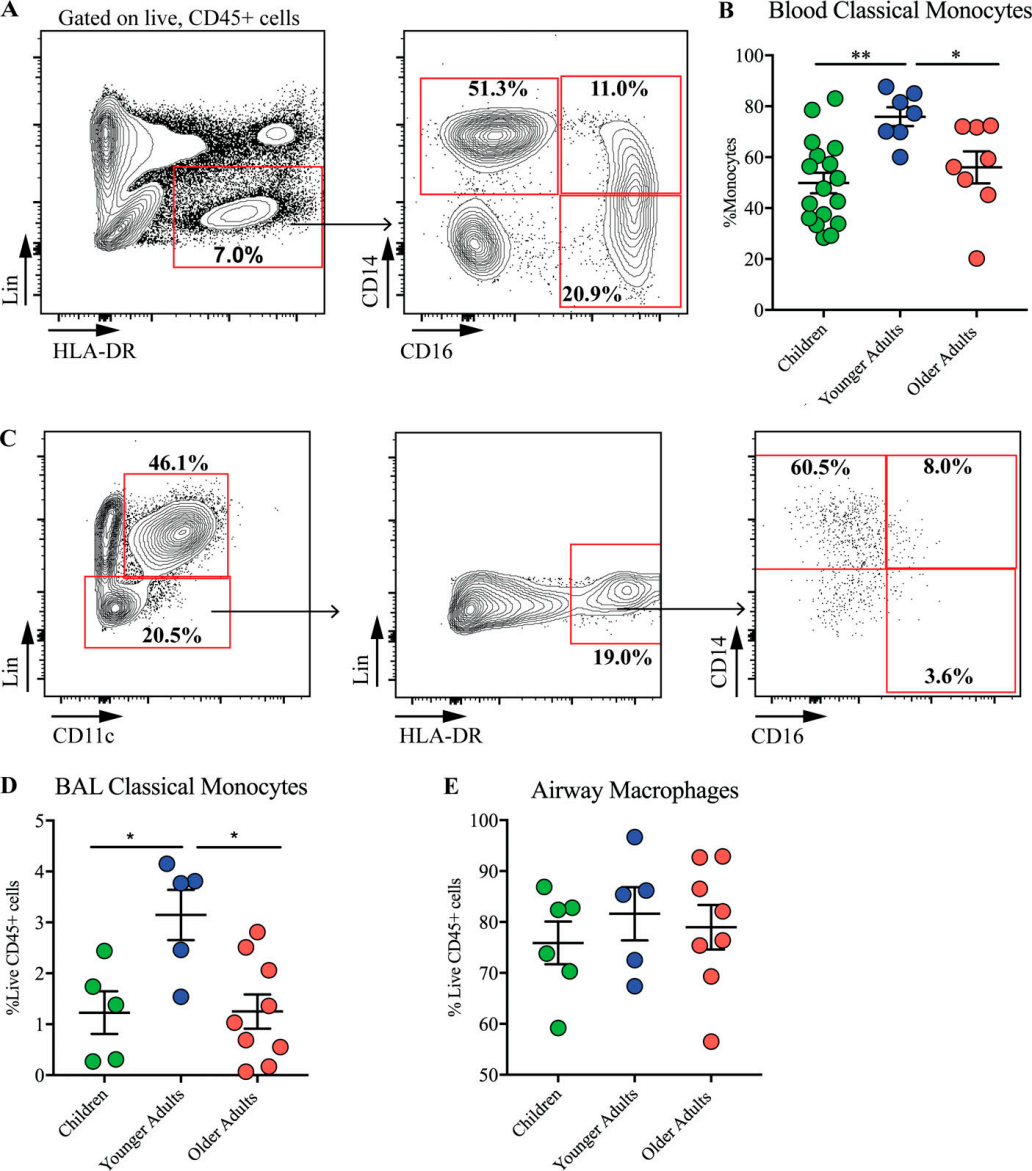
**CM populations in the periphery are similar to those found in the airway. (A)** Gating strategy for polychromatic flow cytometry analysis of blood monocyte subsets. After exclusion of debris and selecting live, CD45^+^ cells, peripheral blood mononuclear phagocytes were identified as Lin^−^ (CD3, CD4, CD8, CD19, CD20, CD34, and FCεRI), HLA-DR^+^cells which were either CD14^+^CD16^− ^(classical monocytes), CD14^+^CD16^+ ^(intermediate monocytes), or CD14^−^CD16^+^ (non-classical monocytes). **(B)** Proportions of classical circulating monocytes in children (*n* = 17), younger adults (*n* = 7), and older adults (*n* = 8). **(C)** Gating strategy for flow cytometry analysis of BAL monocytes. **(D)** Total proportions of CMs in the BAL of children (*n* = 5), younger adults (*n* = 5), and older adults (*n* = 9).****(E)** Proportions of AMs in children (*n* = 6), younger adults (*n* = 5), and older adults (*n* = 8). Values shown are mean ± SEM. *, P < 0.05; **, P < 0.01; Mann–Whitney *U* test.

**Figure S1.**
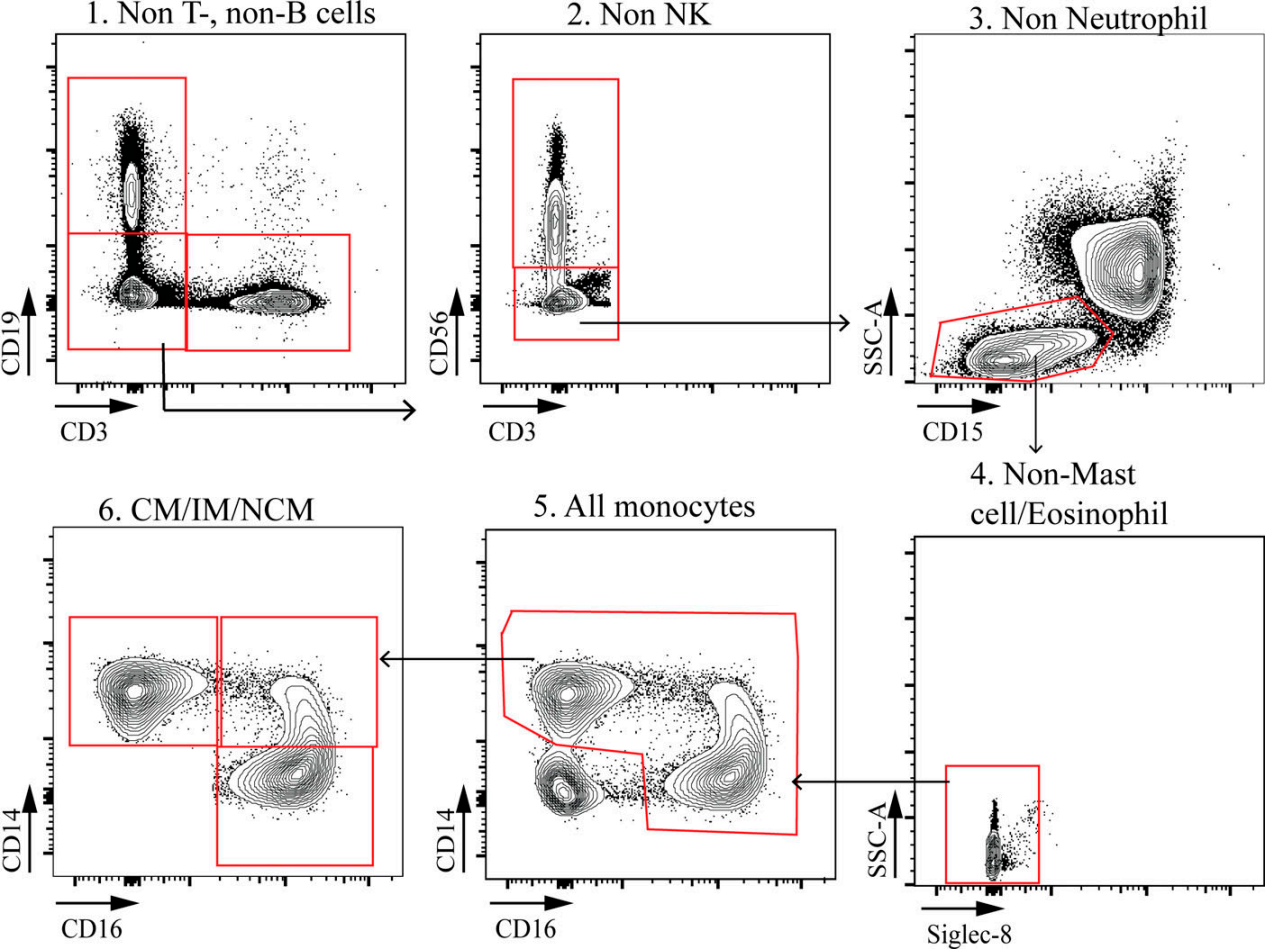
**Gating strategy for polychromatic flow cytometry analysis of peripheral blood CMs in children.** NK, natural killer; SSC, side scatter.

To define circulating and airway monocytes during healthy aging, we used a multicolor flow cytometry gating strategy (Figs. 1 A and S1). After exclusion of debris and doublets, CMs were defined as CD45^+^ cells, negative for lineage markers and positive for CD14 (Patel et al., 2017). In the periphery, proportions of CMs increased in adulthood compared with childhood (ages 2–12 yr; Fig. 1 B); the numbers of these cells then decreased between adult and older age (>50 yr). This pattern of expression was reflected in the airway lumen, with CM populations increasing into adulthood but decreasing with advanced age (Fig. 1, C and D). Proportions of mature AMs were unaffected by age (Fig. 1 E). These data indicate that the circulating monocyte pool is significantly impacted during healthy aging and, furthermore, that the peripheral CM populations are similar to those seen in the airways.

### Comparable patterns of surface protein expression for monocytes found in the airways and periphery

**Figure 2.**
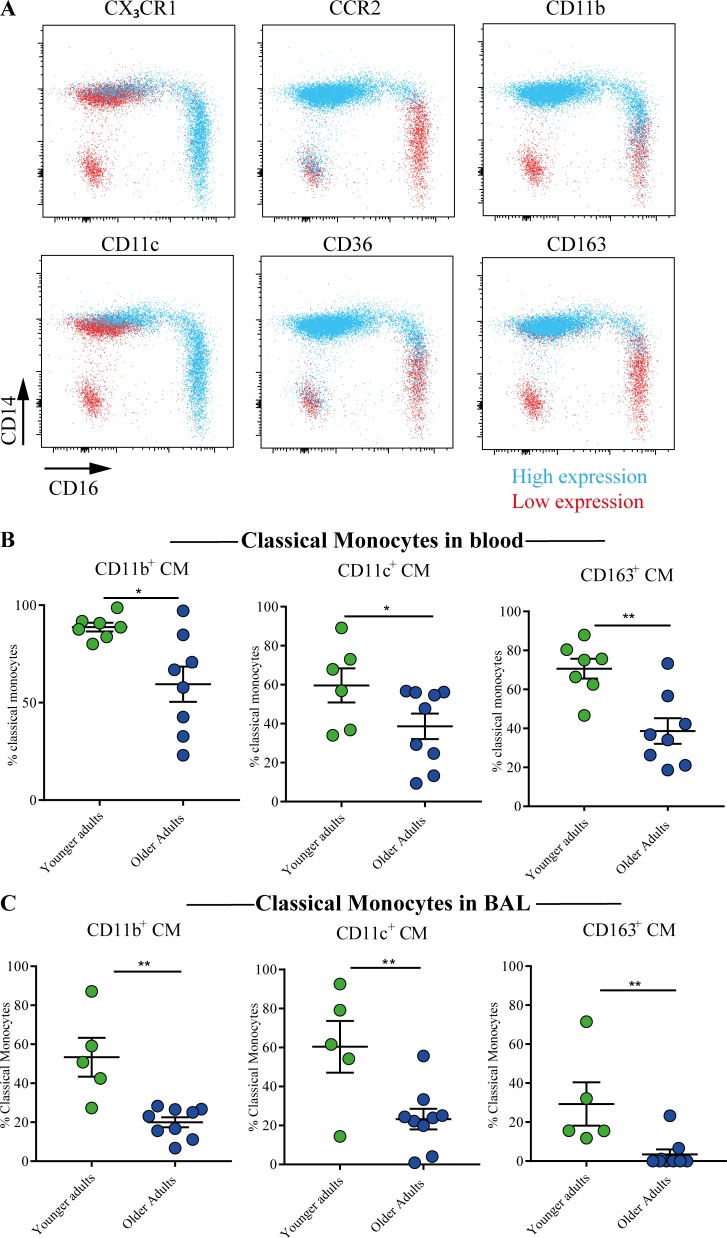
**Comparable patterns of surface protein expression on monocytes found in the airways and periphery. (A)** Back-gating of monocyte phenotypic markers in peripheral blood of healthy controls; blue indicates high expression and red low expression. RNAseq, RNA sequencing. **(B) **Proportions of circulating CMs expressing CD11b in younger adults (*n* = 7) and older adults (*n* = 8), CD11c in younger adults (*n* = 6) and older adults (*n* = 9), and CD163 in younger adults (*n* = 7) and older adults (*n* = 8). **(C)** Proportions of CMs in the BAL of healthy volunteers expressing CD11b, CD11c, and CD163 in younger adults (*n* = 5) and older adults (*n* = 9). Values shown are mean ± SEM. *, P < 0.05; **, P < 0.01, Mann–Whitney *U* test.

**Figure S2.**
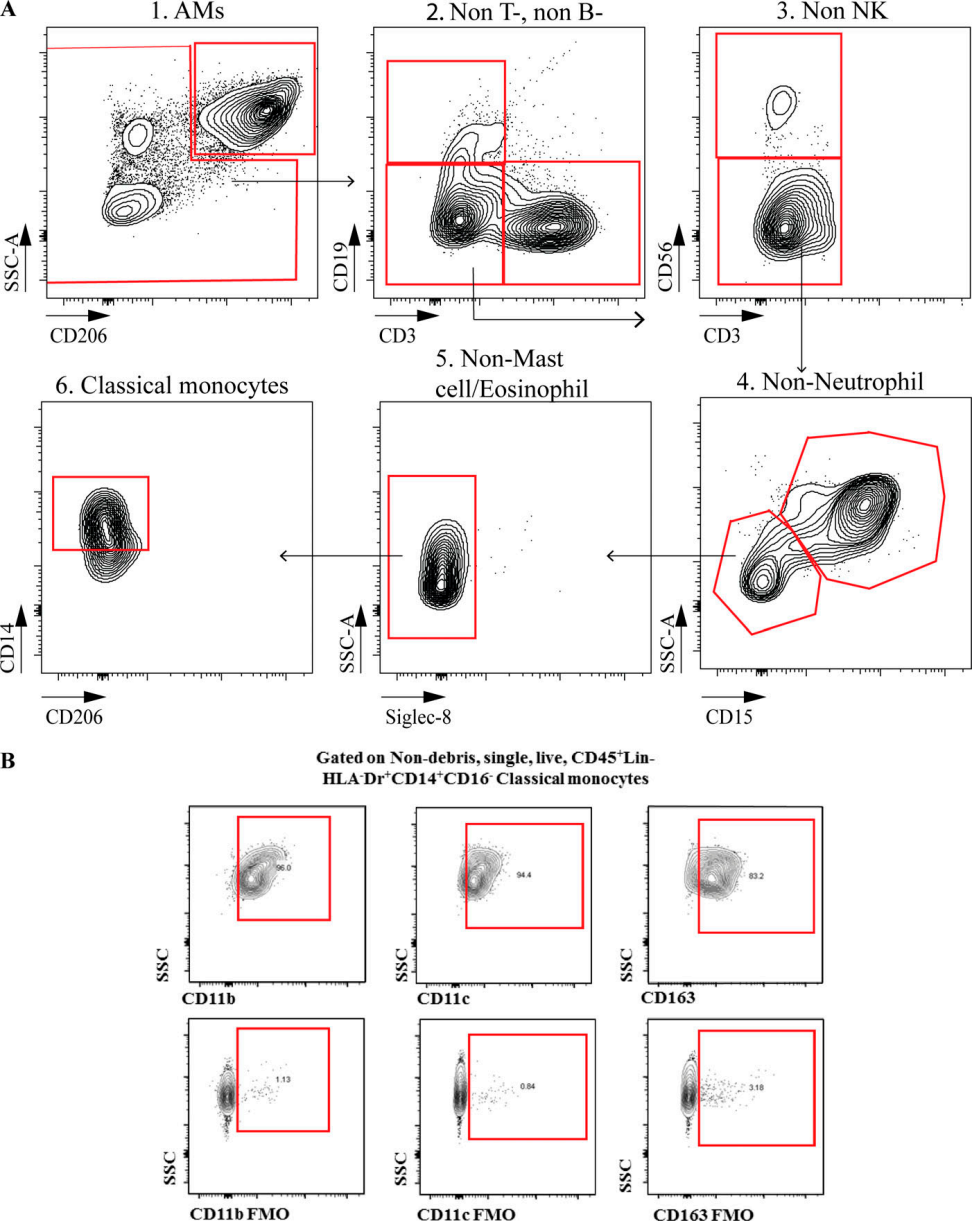
**Flow cytometry analysis of BAL CMs in children.** **(A) **Gating strategy for polychromatic flow cytometry analysis of BAL CMs in children. **(B)** Fluorescence minus one controls for CD11b, CD11c, or CD163 staining. NK, natural killer; SSC, side scatter.

Next, we assessed surface expression of phenotypic markers of monocyte function and activation on CM populations (Figs. 2 A and S2). As cell recovery from BAL is limited in children, we focused on adult populations. The chemokine receptors CCR2 and CX_3_CR1 have previously been shown to be differentially expressed on monocyte subsets (Ziegler-heitbrock et al., 2010; Patel et al., 2017). Consistent with published literature in human systems, CMs expressed high proportions of CCR2 and low levels of CX_3_CR1, while conversely, NCMs expressed low levels of CCR2, concomitant with high proportions of CX_3_CR1 (Fig. 2 A). The β2 integrins CD11b and CD11c are expressed mainly by myeloid cell types and mediate adhesion to the endothelial lining and extracellular matrix components. CD163 is the high-affinity scavenger receptor for the hemoglobin–haptoglobin complex known to be involved in the resolution of inflammation (Tippett et al., 2011). CMs exhibited the highest expression of CD11b and CD163 compared with IMs and NCMs (Figs. 2 A and S2 B). The proportions of peripheral CMs expressing CD11b, CD11c, and CD163 were increased in midadult life compared with older controls (Fig. 2 B). Similar to our findings in total CM populations (Fig. 1) and in the periphery (Fig. 2 B), there was a reduced activation status of BAL CMs with age, characterized by a reduction in the proportions of BAL CMs expressing CD11b, CD11c, and CD163 in older compared with midlife volunteers (Fig. 2 C). CD36 is an 88-kD transmembrane glycoprotein expressed on monocytes that has been shown to function as a scavenger receptor for oxidized low-density lipoproteins (Huh et al., 1996). CD36 was chiefly expressed on CMs, with limited expression on IMs and NCMs; however, its distribution on monocyte populations did not alter with age (Fig. 2 A). Together, these data indicate that ageing significantly impacts circulating monocyte populations and that comparable patterns of expression are found in the airways and periphery.

### AMs in the adult human lung after transplant are peripherally derived

**Figure 3.**
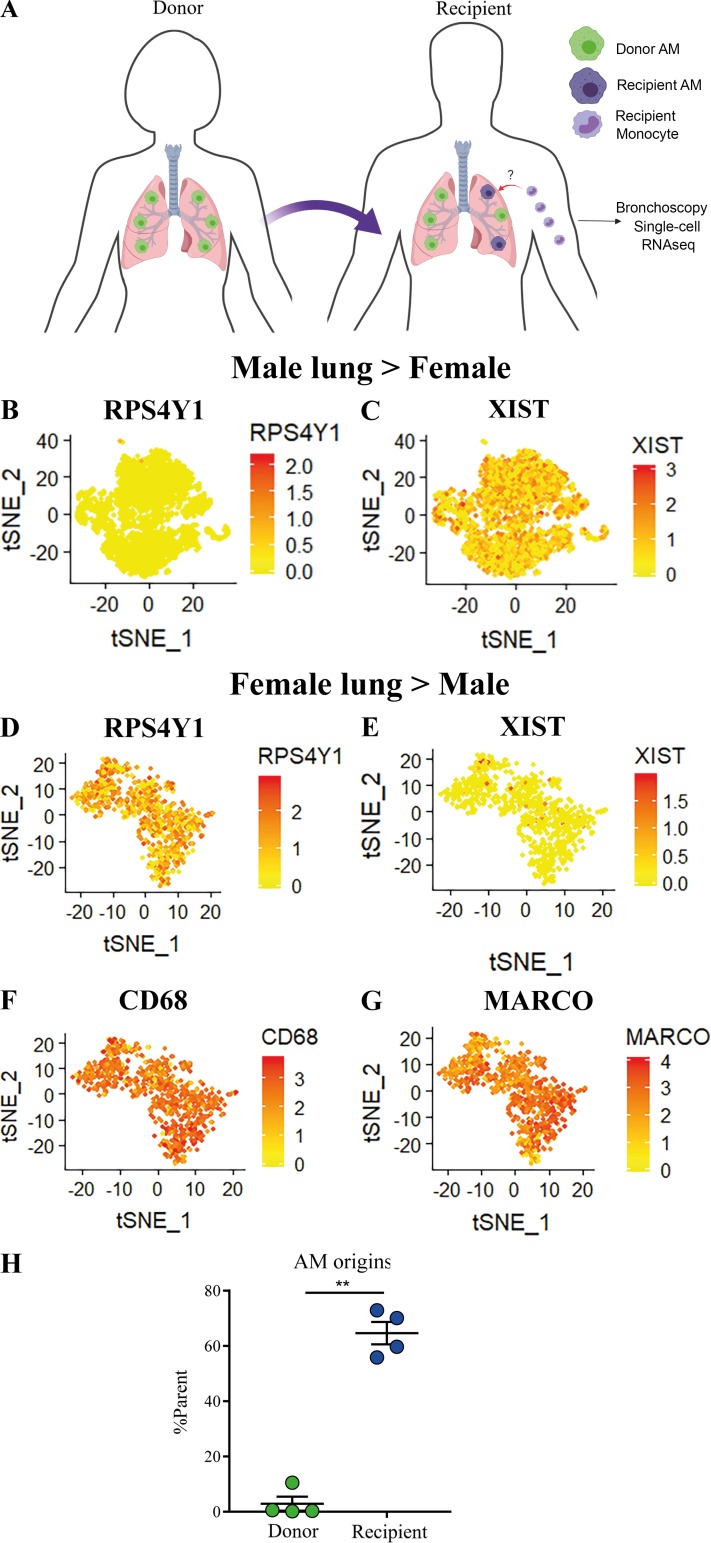
**AMs in the adult human lung after transplant are peripherally derived. (A)** Schematic for sex-mismatched transplant model. Representative *t*-SNE plots showing gene expression levels in single cells derived from sex-mismatched lung transplant patients. **(B)** Male donor to female recipient expressing RPS4Y1. **(C) **Male donor to female recipient expressing XIST. **(D)** Female donor to male recipient expressing RPS4Y1. **(E)** Female donor to male recipient expressing XIST. **(F and G)** Female donor to male recipient expressing CD68 (F) and macrophage receptor with collagenous structure (MARCO; G).** (H) **Quantification of donor- or recipient-derived AMs in lung transplant patients (*n* = 4). Values shown are mean ± SEM. **, P < 0.01, Mann–Whitney *U* test.

Based on murine studies largely conducted using fate-reporter mice, the prevailing opinion is that AMs are seeded during embryonic development and self-maintain independently of peripheral contribution during adulthood (Ginhoux and Guilliams, 2016). Since monocytes are key precursors for mature macrophage populations, we asked whether AMs in the human lung are readily replaced by monocyte-derived cells. To examine the origin of AMs in the human lung, we used BAL cells from sex-mismatched lung transplant recipients (three male→female, one female→male), which were isolated and sorted for live cells (Fig. 3 A). The RNA of single cells was barcoded using 10X Genomics Chromium 3′ single cell solution, amplified, and sequenced. Sequencing data were filtered and aligned to the human reference genome (hg38) and count tables analyzed using Seurat (Butler et al., 2018). Early in development, one X chromosome in female cells is transcriptionally silenced via X-chromosome inactivation in a process known as dosage compensation (Colognori et al., 2019). X-inactive specific transcript (XIST) is encoded in the X-inactivation center of the X chromosome and expressed in female, but not male, somatic cells (Sahakyan et al., 2018). Conversely, ribosomal protein S4 Y-linked 1 (RPS4Y1) is one of the variants encoding the ribosomal protein S4 solely in male somatic cells; XIST and RPS4Y1 have been shown to be robust and tissue-independent sex-specific transcripts (Staedtler et al., 2013). We identified recruited cells as either XIST^+^RPSY41^−^ in the case of a female recipient of a male donor lung (Fig. 3, B and C) or, conversely, XIST^−^RPSY41^+^ in the scenario of a male recipient of a female lung (Fig. 3, D and E). Of four sex-mismatched lung transplant patients, only one male had any AMs of donor origin (17.5%), and this may be due to the relatively recent time since the transplant (8.4 mo). Of note, ∼60% of cells were able to be sex matched (Table 1). Recruited cells highly expressed CD68 (Fig. 3 F) and macrophage receptor with collagenous structure (MARCO; Fig. 3 G), confirming that these cells were mature AM populations. Quantification of recipient AMs showed that the majority of AMs were recipient derived, with minimum contribution from macrophages from the donor organ (Fig. 3 H). These data indicate that after transplant, AMs are replaced by circulating precursors that differentiate into mature peripherally derived AM populations.

Macrophages are critical immune cells and important regulators of inflammatory processes (Byrne et al., 2017). Here, we provide evidence that the majority of AMs in the human lung after transplant are peripherally derived cells rather than resident AMs that originated from the donor. Collectively, our work indicates that the self-renewing population of AMs in the human lung is readily replaced from the peripheral monocyte pool and provides crucial evidence for the origin of AMs that can inform the design of future macrophage-targeted therapies.

Previous studies have reported variations in the proportions and phenotype of monocyte subsets with increasing age (Metcalf et al., 2017; Hearps et al., 2012; Seidler et al., 2010) and in chronic airway diseases such as chronic obstructive pulmonary disease and asthma (Kapellos et al., 2018; Hung et al., 2018). Our study indicates that the circulating monocyte pool dynamically changes during healthy aging and that with increasing age, there is an alteration in peripheral and airway innate immunity. CMs are known to rapidly leave the circulation in response to injury and, upon extravasation into inflamed tissue, differentiate into inflammatory macrophages. We speculate that this might be important in the development and progression of age-related chronic lung diseases such as idiopathic pulmonary fibrosis and chronic obstructive pulmonary disease, through aberrant responses to environmental insults. Since several chronic lung diseases are tightly linked to the aging process, the life span and origins of human AMs have particular relevance for the treatment and diagnosis of such diseases.

Recent mechanistic work in murine models has challenged the importance of the mononuclear phagocyte system in pulmonary immunity. Work using mice that lack hematopoietic stem cells has determined that during prenatal development, fetal liver or yolk sac macrophages give rise to mature macrophages in the lung (Ginhoux and Guilliams, 2016). Hashimoto et al. (2013) used parabiosis and fate-mapping approaches in mice to demonstrate that monocytes do not significantly contribute to lung macrophage populations at homeostasis. Tan and Krasnow (2016) recently showed that macrophage colonization of the lung occurs in sequential waves, with AMs colonizing the airway in the first week of life. Misharin et al. (2017) showed that both monocyte- and perinatally derived AMs were highly activated during fibrotic lung disease and that depletion of peripherally derived AMs after recruitment to the lung ameliorated the severity of fibrosis, whereas depletion of tissue-resident AMs had no effect on the disease. A recent study implicated NCMs in lung allograft rejection in mice, and complementary human data showed the presence of NCMs in human donor lungs before transplant and after perfusion (Zheng et al., 2017). Several studies that have used transplant models to investigate the origins of AMs in the human lung (Bittmann et al., 2001; Eguíluz-Gracia et al., 2016; Nayak et al., 2016; Hunninghake et al., 1980; Thomas et al., 1976). Bittmann et al. (2001) found minimal numbers (∼20%) of donor AMs in the lungs after transplant, which decreased over time. Our research benefits from the combined use of BAL cells in suspension (as opposed to biopsies which have inherent issues, including antibody/probe staining in sections, loss of airway cells upon sectioning, and potential loss of compartmentalization) and sex mismatching (rather than HLA mismatching, which has inherent issues of AM activation) coupled with the power of single-cell sequencing. Our findings indicate that the overwhelming majority of AMs in the lung after transplant are recruited from monocyte precursors and questions a model where prenatally derived AMs persist in the human lung over a lifetime. Indeed, the human lung is exposed to multiple antigenic and environmental triggers on a daily basis and over a much longer timeframe than can be investigated in murine studies where mice are housed in a sterile environment. Our work further indicates that the majority of donor AMs failed to self-maintain and were replaced by AMs originating from the peripheral circulation of the transplant recipient; this raises the question of the kinetics of this process in the human lung. Of note, the most recent lung recipient in our study was the only patient exhibiting even a small number of AMs of donor origin, suggesting that replacement is completed within a year in this context. Ideally, studies that track AM populations longitudinally would address this question but may be challenging clinically.

In summary, our data show that during healthy aging, there are distinct alterations in circulating and airway monocyte populations and, furthermore, that the majority of AMs in the human airspace after transplant are derived from circulating monocytes. We speculate that AM populations may be considerably more dynamic and less stable than murine ontogeny studies imply, underlining the importance of studies that attempt to delineate the origins of AMs in human systems. Our data emphasize the importance of AMs of hemopoietic origin in the human lung and suggest that these cells may represent a novel target for future treatment of age-related chronic lung disease.

## Materials and methods

### Collection of BAL samples

BAL from healthy adult volunteers was obtained as previously described (Molyneaux et al., 2017; Allden et al., 2019). Briefly, bronchoscopies were performed under a light sedation with midazolam in combination with local anesthesia with lidocaine. Four 60-ml aliquots of warmed sterile saline were instilled in to the right middle lung lobe and aspirated by syringe. Lavage aliquots collected after each instillation were pooled for each subject. Volume and BAL appearance was recorded for all samples. All adult subjects provided written, informed consent to participate in the study. Pediatric controls were all undergoing clinically indicated bronchoscopy to investigate upper airway symptoms as previously described (Bossley et al., 2012; Saglani et al., 2007). None of the children had lower airway symptoms. Parental written informed consent and age-appropriate child assent was obtained. Studies were approved by National Research Ethics Committees (15/LO/1399 [adult subjects] or 15/LO/1885, 08/H0708/3, and 15/SC/0569 [children]).

### BAL processing

BAL samples were processed and stained on the day of sample collection. Whole BAL was strained through a 70-µM sterile strainer and subsequently centrifuged (700 ×*g*, 5 min, 4°C) and pellets subjected to red blood cell lysis (155 mM NH_4_Cl, 10 mM KHCO_3_, and 0.1 mM EDTA, pH 7.3) for 10 min before washing and resuspension in complete media (RPMI + 10% fetal calf serum, 2 mM L-glutamine, and 100 U/ml penicillin/streptomycin).

### Peripheral blood mononuclear cell isolation

Blood was obtained, following consent, by venipuncture from healthy volunteers. Human peripheral blood mononuclear cells were isolated using Percoll density gradient centrifugation, as per the manufacturer’s instructions.

### Flow cytometry

Cells were washed and incubated with near-infrared fixable live/dead stain (Life Technologies), as per the manufacturer’s instructions. Cells were washed before incubation with human Fc Block (BD Pharmingen), and surface staining was performed with a lineage cocktail of the following antibodies (clones in parentheses) purchased from BioLegend: CD3 (HIT3a), CD4 (OKT4), CD8 (HIT8a), CD19 (HIB19), CD20 (2H7), CD34 (561), FcER1 (AER-37), CD45 (HI30), CX_3_CR1 (2A9-1), CCR2 (K036C2), CD36 (5–271), CD11b (M1/70), CD11c (3.9), CD16 (B73.1), CD163 (GHI/61), CD14 (M5E2), CD206 (15–2), CD86 (IT2.2), CSFR1 (9-4D2-1E4; BD Biosciences), and HLA-DR (G46-6). Total macrophage populations were identified as auto-fluorescent, CD11c^+^ cells. Surface staining was followed by fixation and then permeabilization to allow for intracellular or intranuclear staining. Labeled cells were acquired on a fluorescence-activated cell sorter (LSR Fortessa II; BD Bioscience) and further analyzed using FlowJo (Tree Star).

### BAL preparation for single-cell RNA sequencing

Cryopreserved BAL cells were thawed and viable cells sorted on a BD Influx cell sorter (Becton Dickinson) using propidium iodide into Dulbecco’s PBS + 0.04% bovine serum albumin and retained on ice. Sorted cells were counted and assessed for viability with Trypan Blue using a Countess automated counter (Invitrogen) and then resuspended at a concentration of 800–1,000 cells/µl.

### Single-cell RNA sequencing

Single-cell suspensions were loaded onto 10X Genomics Single Cell 3′ Chips along with the RT mastermix as per the manufacturer’s protocol for the Chromium Single Cell 3′ Library (v2, PN-120233; 10X Genomics), to generate single-cell gel beads in emulsion. RT was performed using a C1000 Touch Thermal Cycler with a Deep Well Reaction Module (Bio-Rad) as follows: 55°C for 2 h; 85°C for 5 min; hold 4°C. cDNA was recovered and purified with DynaBeads MyOne Silane Beads (catalog no. 37002D; Thermo Fisher Scientific) and SPRIselect beads (catalog no. B23318; Beckman Coulter). Purified cDNA was amplified as follows: 98°C for 3 min; 12 times (98°C for 15 s, 67°C for 20 s, 72°C for 60 s); 72°C for 60 s; hold 4°C. Amplified cDNA was purified using SPRIselect beads and sheared to ∼200 bp with a Covaris S2 instrument using the manufacturer’s recommended parameters. Sequencing libraries were generated with unique sample indices for each sample. Libraries for samples 1–3 and 4–5 were multiplexed respectively and sequenced on an Illumina NextSeq 500 (NextSeq control software v2.0.2/ Real Time Analysis v2.4.11) using a 150-cycle NextSeq 500/550 High Output Reagent Kit v2 (FC-404-2002; Illumina) in stand-alone mode as follows: 98 bp (read 1), 14 bp (I7 index), 8 bp (I5 index), and 485 10 bp (read 2).

### Bioinformatics

Processing of the sequencing data into transcript count tables was performed using the Cell Ranger Single Cell Software Suite by 10X Genomics (v2.0.0). Raw base call files from the NextSeq 500 sequencer were demultiplexed, using the Cell Ranger mkfastq pipeline, into sample-specific FASTQ files. These FASTQ files were then processed with the Cell Ranger count pipeline, where each sample was processed independently. First, Cell Ranger count used STAR (Dobin et al., 2013) to align cDNA reads to the GRCh38 human reference transcriptome, which accompanied the Cell Ranger Single Cell Software Suite. Aligned reads were filtered for valid cell barcodes and unique molecular identifiers (UMIs), and observed cell barcodes were retained if they were 1 Hamming distance away from an entry in a whitelist of known barcodes. UMIs were retained if they were not homopolymers and had a quality score >10 (90% base accuracy). The Cell Ranger count corrected mismatched barcodes if the base mismatch was due to sequencing error, as determined by the quality of the mismatched base pair and the overall distribution of barcode counts. A UMI was corrected to another more prolific UMI if it was 1 Hamming distance away and shared the same cell barcode and gene. The Cell Ranger count examined the distribution of UMI counts for each unique cell barcode in the sample and selected cell barcodes with UMI counts that fell within the 99th percentile of the range defined by the estimated cell count value. The expected cell count value of 10,000 was used for this experiment. Counts that fell within an order of magnitude of the 99th percentile were also retained. The resulting data for each sample were then aggregated using the cellranger aggr pipeline, followed by a between-sample normalization step. After aggregation, the count data were processed and analyzed using a comprehensive pipeline assembled and optimized in-house as described below.

To preprocess the mapped data, we constructed a cell quality matrix based on the following data types: library size (total mapped reads), total number of genes detected, percentage of reads mapped to mitochondrial genes, and percentage of reads mapped to ribosomal genes. Cells that had any of the four parameter measurements higher than three times the median absolute deviation of all cells were considered outliers and removed from subsequent analysis.

Downstream analyses were performed using the R software package Seurat (Butler et al., 2018). This involved filtering to remove mitochondrial reads >20% or ribosomal reads >50%. To exclude genes that were potentially detected from random noise, we removed genes that were detected in <1% of all cells. Before normalization, abundantly expressed ribosomal protein genes and mitochondrial genes were discarded to minimize the influence of those genes in driving clustering and differential expression analysis.

Subsequent analysis included dimensional reduction of data using principal component followed by *t*-distributed stochastic neighbor embedding (*t*-SNE) algorithm for modularity-driven clustering, based on a cell–cell distance matrix constructed on these principal components. Expression greater than zero was used for sex cell determination with XIST for female cells and RPS4Y1 for male cells. *t*-SNE plots with expression levels for genes were generated using the FeaturePlot function in Seurat (Butler et al., 2018).

### Statistics

Differences between noncontinuous groups were compared using a Mann–Whitney *U* test. To explore associations between pairs of continuous variables, Spearman’s rank correlation was used. Analysis was performed using GraphPad Prism and Spotfire software. All tests were two sided, and P < 0.05 was considered to be statistically significant.

### Online supplemental material

Fig. S1 shows the flow cytometry gating strategy used for the analysis of peripheral blood CMs. Fig. S2 shows the flow cytometry gating strategies used for the analysis of BAL CMs and representative fluorescence minus one controls for CD11b, CD11c, or CD163 staining.
